# Hydrogel Vehicles for Enteric-Coated Pantoprazole Minitablets: Impact of Polymer Type on Rheology and Drug Release

**DOI:** 10.3390/gels12060526

**Published:** 2026-06-11

**Authors:** Maja Frankiewicz, Katarzyna Centkowska, Barbara Kwiecien, Kinga Maksymowicz, Justyna Dobosz, Michal Smolenski, Marcela Staniszewska, Jadwiga Paszkowska, Grzegorz Garbacz, Malgorzata Sznitowska

**Affiliations:** 1 Department of Pharmaceutical Technology, Medical University of Gdansk, Hallera 107, 80-416 Gdansk, Poland; maja.frankiewicz@gumed.edu.pl (M.F.); katarzyna.centkowska@gumed.edu.pl (K.C.); barbara.kwiecien@gumed.edu.pl (B.K.); kmaksymowicz@gumed.edu.pl (K.M.); 2 Physiolution, Pilsudskiego 74/51B, 50-020 Wroclaw, Poland; j.dobosz@physiolution.pl (J.D.); m.smolenski@physiolution.pl (M.S.); m.staniszewska@physiolution.pl (M.S.); j.paszkowska@physiolution.pl (J.P.); g.garbacz@physiolution.pl (G.G.)

**Keywords:** hydrogels, pantoprazole, minitablets, hypromellose, carbomer, sodium alginate, rheology, drug release, pediatric formulation, gastro-resistant formulations

## Abstract

The development of age-appropriate pediatric dosage forms remains an important challenge, particularly for acid-labile drugs requiring gastro-resistant protection. Pantoprazole, a proton pump inhibitor, must be protected from gastric acid until intestinal absorption; however, conventional enteric-coated tablets may be difficult to use in younger children, while manipulation of dosage forms or mixing with food can compromise dose accuracy and drug release performance. Multiparticulate systems, such as minitablets, pellets, or granules, offer flexible dosing but may still require a suitable vehicle to improve acceptability, handling, and ease of swallowing. In this study, enteric-coated pantoprazole minitablets were developed and evaluated after dispersion in selected hydrogel vehicles intended to serve as standardized alternatives to food-based carriers. Hydrogels based on hypromellose (HPMC), carbomer (CAR), and sodium alginate (SA) were characterized in terms of pH, rheological properties, firmness, acid penetration, and their effect on pantoprazole release. Dissolution performance was assessed using both conventional pharmacopoeial testing and dynamic non-pharmacopoeial conditions. Low-concentration gels prepared from high-viscosity HPMC grades showed the most favorable performance, combining suitable spoonable consistency with limited impact on drug release. Among them, 5% HPMC 65SH4000 was particularly promising, as it did not markedly delay pantoprazole release in either pharmacopoeial or dynamic dissolution testing. CAR gels provided advantageous rheological properties, including high viscosity at rest and shear-thinning behavior, and allowed efficient pantoprazole release after transition to buffer conditions; however, their interaction with enteric-coated minitablets should be further optimized with respect to gel amount, concentration, and neutralization strategy. SA gel showed strong structural persistence and delayed release under pharmacopoeial conditions, although this effect was less pronounced in the dynamic model. Overall, the findings indicate that appropriately selected hydrogels may improve the practical use of pediatric multiparticulate formulations, but their composition, pH, rheology, and interaction with enteric coatings must be carefully evaluated.

## 1. Introduction

Oral gels are increasingly recognized as valuable pharmaceutical vehicles for improving the administration of solid oral dosage forms, particularly in patient groups with swallowing difficulties or special dosing needs. Their semisolid consistency, tunable rheological properties, and potential for taste masking make them attractive carriers for pediatric and geriatric drug delivery [1]. When patients are unable to swallow solid dosage forms intact, medicines are commonly crushed and mixed with food to facilitate administration. Such manipulation of the dosage form may lead to dosing errors and unwanted interactions with the co-administered vehicle. In contrast to this conventional practice, oral gels may provide a more standardized and pharmaceutically controllable vehicle. For enteric formulations, mechanical manipulation of the dosage form is particularly inappropriate, as disruption of the coating may compromise gastro-resistance and alter the intended release profile. This issue is highly relevant for pantoprazole, a proton pump inhibitor (PPI) widely used in the treatment of peptic ulcer disease and gastroesophageal reflux disease (GERD). Both conditions are associated with gastric acid-mediated mucosal injury and are frequently managed with acid-suppressive therapy. Pantoprazole, like other PPIs, is an acid-labile prodrug. It must therefore be protected from gastric acid, absorbed in the intestine, and subsequently activated in the acidic secretory canaliculi of gastric parietal cells, where it irreversibly inhibits the H^+^/K^+^-ATPase proton pump [2]. Consequently, premature exposure to gastric acid leads to drug degradation and loss of activity, which necessitates the use of gastro-resistant oral dosage forms.

Pantoprazole was selected as the model drug because it combines high clinical relevance with a demanding formulation profile. Pantoprazole is used in pediatric pharmacotherapy, including children from 1 year of age, while the availability of age-appropriate formulations remains limited and differs between markets [3]. Therefore, there remains a practical clinical need for flexible, acceptable, and gastro-resistant oral dosage forms that can be administered without crushing or otherwise manipulating the enteric-protected drug product.

Minitablets (MTs) represent a promising strategy in this context. Owing to their small size, typically 1–3 mm in diameter, they enable flexible dosing by varying the number of administered units and may be more acceptable for children than conventional tablets or liquid formulations [4]. In addition, minitablets can be enteric-coated, making them suitable carriers for acid-sensitive drugs such as pantoprazole [5]. However, the administration of multiple MTs may be inconvenient and may increase the risk of dosing errors or loss of units during handling.

One possible solution is the use of oral hydrogels as administration vehicles. Hydrogels may facilitate swallowing, reduce the perception of solid particles, improve palatability, and eliminate the need for co-administration with food [6]. Importantly, such gel vehicles could be useful not only for MTs but also for other enteric multiparticulates, including pellets and granules. However, when oral gels are used as vehicles for enteric-coated MTs, their effect on the integrity of the coating and subsequent drug release must be carefully assessed. For acid-labile compounds such as pantoprazole, even subtle formulation–vehicle interactions may be pharmaceutically relevant [7].

Although previous studies have explored the acceptability of pediatric multiparticulate dosage forms [4,8,9], and other studies have investigated hydrogels as drug-loaded or stimuli-responsive delivery systems [10], less attention has been given to the use of simple oral hydrogels as standardized administration vehicles for enteric-coated multiparticulates containing acid-labile drugs. This distinction is important because, in such systems, the hydrogel is not intended to act as the primary drug-release matrix, but rather as an administration aid that must improve handling and swallowability without compromising gastro-resistance or delaying intestinal drug release. The novelty of the present study therefore lies in the integrated evaluation of hydrogel vehicles for enteric-coated pantoprazole MTs, combining rheological and application-related characterization with acid-penetration assessment, pharmacopoeial dissolution testing, and dynamic AMP testing under pH-transition and mechanical-stress conditions.

Against this background, the combination of enteric-coated pantoprazole MTs with oral gel vehicles appears to be a promising approach for developing flexible and patient-friendly pediatric dosage forms. From a regulatory perspective, hydrogels are not specifically identified by EMA or FDA guidance as a separate category of administration vehicles. However, their use is consistent with the broader expectation that pediatric oral medicines should be age-appropriate, acceptable, and supported by justified administration strategies. In this context, hydrogels may be considered pharmaceutically controllable alternatives to non-standardized food-based carriers, provided that their composition, pH, rheological properties, compatibility with the dosage form, and impact on drug release are experimentally verified [11,12].

Therefore, the aim of the present study was to develop enteric-coated pantoprazole MTs and to evaluate their quality and dissolution performance after dispersion in selected oral hydrogel vehicles. Hydrogels based on hypromellose, carbomer, and sodium alginate were selected as representative pharmaceutically established gel-forming polymers with different rheological behavior and pH responsiveness. The study focused on identifying vehicles with suitable handling and spoon-dosing properties that would preserve the gastro-resistant function of the enteric coating and allow appropriate pantoprazole release after transition to intestinal conditions. To address these aspects, the formulations were evaluated using physicochemical and rheological characterization, acid-penetration testing, conventional pharmacopoeial dissolution testing, and dynamic AMP dissolution conditions simulating pH transition and mechanical stress during gastrointestinal transit.

## 2. Results and Discussion

### 2.1. Physicochemical Characterization of Uncoated Minitablets

Pantoprazole is known to be highly susceptible to heat and moisture, which complicates both direct compression and the coating process. Therefore, a granulation step using a 25% *w*/*w* sodium carbonate solution was introduced to improve pantoprazole stability [13]. As a result, the compression process proceeded without complications, and 3 mm MTs containing 25% *w*/*w* of the pantoprazole were successfully manufactured. The obtained MTs complied with European Pharmacopoeia (Ph. Eur. 12.1; 2.9.5) requirements for mass uniformity (22.5 mg ± 10%) and exhibited favorable mechanical properties [14]. Their mean hardness was 25.8 ± 2.71 N, while friability remained low at 0.1%. Adequate mechanical strength of the cores was essential to ensure successful fluid-bed coating.

### 2.2. Physicochemical Characterization of Enteric-Coated Minitablets

The enteric film was separated from the pantoprazole cores by applying a hypromellose (HPMC) sub-coating, as interactions may occur between acid-sensitive pantoprazole and enteric polymers containing acidic groups [15]. The film thickness of the HPMC sub-coating was approximately 37 µm. The MTs were subsequently coated with Eudragit L 30 D-55. During the coating process, no major technological problems were observed. The mean thickness of the enteric coat was 63.4 ± 5.5 µm. In the disintegration test, the coated MTs remained intact after 2 h in 0.1 mol/L HCl, with no visible signs of film damage. After transfer to phosphate buffer (pH 6.8), disintegration occurred within 12 min. These results indicate good resistance of the coated MTs to acidic conditions and rapid disintegration at intestinal pH, confirming compliance with pharmacopeial requirements for enteric dosage forms.

### 2.3. Physicochemical Characterization of Oral Gel Vehicles

Hydrogels were prepared using commonly applied hydrophilic gel-forming polymers, including carbomer (CAR), sodium alginate (SA), and four grades of HPMC: 606, 60SH4000, 65SH4000, and 90SH4000. The HPMC grades selected for this study represented different substitution types and viscosity grades. HPMC is available in different substitution types, defined by the relative content of methoxy and hydroxypropoxy groups, which may influence polymer hydration, gel-network formation, water distribution, and interaction with the dissolution medium. In the present study, three high-viscosity HPMC grades with comparable nominal viscosity but different substitution types were selected: HPMC 60SH4000, HPMC 65SH4000, and HPMC 90SH4000, corresponding to types 2910, 2906, and 2208, respectively [Ph. Eur. 12.1; Hypromellose monograph]. This selection allowed the effect of substitution type to be evaluated under conditions of comparable nominal viscosity. In addition, HPMC 606 was included as a low-viscosity grade to assess the effect of viscosity grade and polymer concentration on gel properties and pantoprazole release. All tested hydrogels and their polymer concentrations are listed in Table 1. All polymers, except HPMC 606, formed semi-solid gels at concentrations below 6%. In contrast, the low-viscosity HPMC 606 required substantially higher concentrations, 20–22%, to achieve an adequate consistency suitable for spoon dosing. The target viscosity range was selected on the basis of macroscopic evaluation and rheological testing, with particular attention to the possibility of administering the gels with a teaspoon and facilitating the swallowing of co-administered MTs.

The pH of the hydrogels was within the values between 4.2 and 7.8. The HPMC SH-grade gels showed a lower pH between 4.2 and 5.3, while HPMC 606 and SA gels had pH values close to neutral. CAR gels were neutralized with sodium hydroxide (10% *w*/*w* NaOH) or tris(hydroxymethyl)aminomethane (20% *w*/*w* TRIS) to pH values between 6.0 and 7.0. Neutralization of CAR leads to ionization of carboxyl groups, polymer-chain expansion, swelling, and formation of a structured microgel network. Overall, the pH values of the developed formulations were within or close to the range generally considered suitable for oral liquid preparations. From the perspective of oral administration, pH values close to neutral may be preferable for patient comfort and palatability, particularly in pediatric use. In contrast, the mildly acidic pH of selected HPMC SH-grade gels may contribute to sour taste, reduced acceptability, or oral discomfort, and should therefore be considered during further formulation optimization. Although the intended use of these gels involves immediate administration and a short residence time in the oral cavity, repeated exposure to acidic preparations may raise additional oral-safety considerations, including potential effects on tooth enamel. Therefore, the addition of suitable pH-adjusting excipients may be considered to increase the pH of acidic gel formulations; however, such a modification would require careful evaluation because changes in pH may also influence the integrity and dissolution behavior of the enteric coating [16].

The viscosity–shear rate curves showed that all gels exhibited shear-thinning (non-Newtonian behavior) as their apparent viscosity decreased with increasing shear rate. At a shear rate of 100 s^−1^, the apparent viscosity of the SA and HPMC hydrogels at 25 °C ranged from approximately 2.8 to 20 Pa·s, whereas at 0.01 s^−1^ it ranged from approximately 16 to 360 Pa·s, depending on polymer type and concentration (Table 1).

For SA, the apparent viscosity of the 4% gel was comparable to that of 4% HPMC 4000-grade gels and was approximately 7 Pa·s at 100 s^−1^. However, the SA gel showed only a modest difference between near-rest conditions, measured at 0.01 s^−1^, and shear conditions, measured at 100 s^−1^. SA gel showed comparable viscosity values at a shear rate of 100 s^−1^, corresponding to flow behavior, both at room temperature (25 °C; application temperature) and at human body temperature (37 °C). SA is a natural linear polysaccharide composed of β-D-mannuronic acid (M) and α-L-guluronic acid (G) residues arranged in different sequences and blocks. Its gelation behavior depends on the M/G ratio and block distribution and may involve intermolecular associations, such as electrostatic interactions, hydrogen bonding and van der Waals forces, resulting in a weak reversible network. Its gelation behavior depends strongly on environmental conditions. In the presence of divalent cations (e.g., Ca^2+^), SA forms ionic gels according to the “egg-box” model, whereas under acidic conditions protonation of carboxyl groups may lead to acid-induced gelation or precipitation [17]. Therefore, although SA is widely used as a biocompatible gelling polymer, ion- and pH-dependent structural changes may be disadvantageous for oral co-administration with enteric-coated MTs, particularly under gastric and intestinal pH-transition conditions [10]. Potential batch-to-batch variability should be considered, particularly for natural polymers such as sodium alginate, whose rheological behavior may depend on molecular weight, M/G ratio, block distribution, purity, and ionic composition. The present study assessed the within-batch repeatability of the tested formulations, but the inter-batch variability of polymer raw materials was not evaluated. Further development of alginate-based vehicles would therefore require confirmation of batch-to-batch consistency in rheological properties, acid responsiveness, and dissolution performance.

The 4% HPMC gels had similar apparent viscosity values, approximately 7 Pa·s at 100 s^−1^, irrespective of the pharmacopoeial substitution type, namely 2910, 2906, or 2208. Increasing the HPMC concentration by 1% resulted in an approximately two-fold, or even greater, increase in apparent viscosity, indicating a strong concentration-dependent thickening effect. Only for the low-viscosity HPMC 606 grade was it necessary to use a markedly higher polymer concentration, namely 20%, to obtain a consistency comparable to that achieved with the high-viscosity HPMC 60SH, 65SH, and 90SH grades.

Aqueous HPMC gels are known to exhibit inverse thermoreversible gelation, characterized by a sol–gel transition upon heating and a reversible gel–sol transition upon cooling. However, under the rheological conditions used in the present study, no substantial differences in viscosity were observed between 25 °C and 37 °C, suggesting that the tested temperature range was below the threshold required for a high-viscosity polymer to induce pronounced thermal gelation [18]. Despite its high polymer concentration, HPMC 606 formed gels with low viscosity and only minor shear-rate-dependent changes, suggesting weak intermolecular interactions and a poorly developed internal network. This may be related to the predominance of shorter polymer chains, which increase polymer content but are less effective in forming an extended three-dimensional structure. The marked increase in viscosity observed at elevated temperature may reflect enhanced polymer–polymer interactions and the onset of thermally induced gelation [19].

The weak internal network structure of HPMC 606 gels was also supported by the texture analysis results. The low firmness of HPMC 606 gels (Table 1) despite their high polymer concentration is most likely related to the low-viscosity character of this grade. HPMC 606 contains shorter polymer chains than high-viscosity HPMC grades, which limits chain entanglement and formation of a mechanically coherent gel network. Therefore, the increased polymer concentration raised the solids content but did not translate into proportionally higher firmness. In contrast, most gels prepared with other HPMC grades, including 60SH, 65SH and 90SH, as well as CAR gels, showed higher firmness values. Even at lower concentrations, such as 4% HPMC or 0.5% CAR, firmness values were approximately 150–160 g, indicating a more developed and mechanically resistant gel structure.

For carbomer gels, a pronounced shear-thinning behavior was observed, characterized by very high apparent viscosity at low shear and a substantial decrease in viscosity after exceeding the yield-stress region. The apparent viscosity values (Table 1), measured at a low shear rate of 0.01 s^−1^ under steady-state conditions at 25 °C, were very high: approximately 1520–1660 Pa·s for 0.5% CAR and 2320–2400 Pa·s for 1% CAR. The observed differences in viscosity between CAR gels neutralized with NaOH and TRIS were within the range of SD and were therefore considered not relevant. At higher shear rates, after exceeding the yield-stress region, CAR gels displayed the lowest viscosity among the tested gels, reaching approximately 2.0 and 3.5 Pa·s at 100 s^−1^ for 0.5% and 1% CAR, respectively. This combination of high viscosity at rest and low viscosity under shear may be advantageous for oral administration, as the gel remains cohesive on a spoon but becomes easier to spread and swallow during administration.

Gels prepared from CAR showed comparable viscosity values at a shear rate of 100 s^−1^, representative of flow conditions, both at room temperature (25 °C; application temperature) and at human body temperature (37 °C). This finding is consistent with literature data indicating that CAR gels are generally highly stable under temperature variations; even brief exposure to elevated temperatures, such as during sterilization, does not substantially alter their formulation characteristics [20]. However, under very low-shear conditions, a lower apparent viscosity was observed at 37 °C. This difference should be interpreted cautiously, as it may reflect the highly non-linear rheological response of CAR gels in the low-shear or yield-stress region rather than a simple temperature-driven decrease in viscosity [21,22].

**Figure 1:**
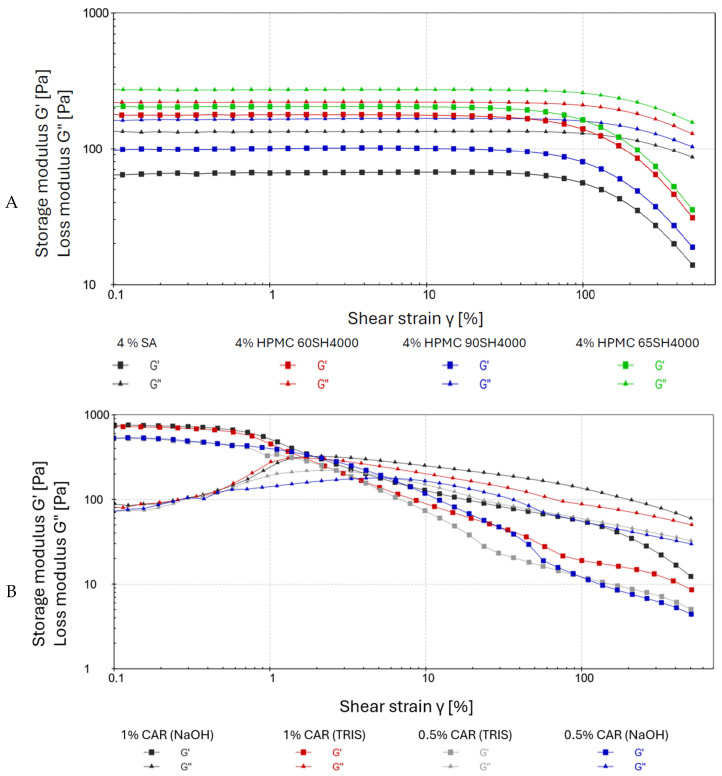
Amplitude sweep profiles of selected hydrogels: (**A**) 4% HPMC 60SH4000, 65SH4000, 90SH4000, and SA; (**B**) 0.5% and 1% CAR gels at an angular frequency of ꙍ = 10 rad/s.

To further characterize the mechanical strength and yielding behavior of the developed gels, stress amplitude sweep tests were also performed, and the results are presented in Figure 1. SA and HPMC gels, irrespective of HPMC substitution type within the studied concentration range, did not show a distinct yield point within the tested stress range, as indicated by the absence of a crossover between the storage modulus (G′) and loss modulus (G″) (Figure 1A). Together with the viscosity profiles, this supports their classification as shear-thinning systems without a pronounced yield point. Only the CAR gels, irrespective of polymer concentration (0.5% or 1%) and neutralizing agent (NaOH or TRIS), exhibited a flow stress (τᵧ), defined as the crossover point at which G′ = G″. The τᵧ values increased with polymer concentration and were consistently higher for TRIS-neutralized gels than for NaOH-neutralized gels. The values were 220.9 Pa and 177.6 Pa for 0.5% CAR gels neutralized with TRIS and NaOH, respectively, and 309.5 Pa and 258.6 Pa for the corresponding 1% CAR gels (Figure 1B). The yield-stress nature of CAR gels can be attributed to the presence of high-molecular-weight, crosslinked polyacrylate chains forming a swollen microgel network with interparticle interactions and chain entanglements, which prevent flow at low shear stresses [23]. Literature data show that CAR gels at concentrations above approximately 0.7–1.0% undergo a transition from viscous liquid-like behavior to a densely jammed, solid-like state, accompanied by the development of a pronounced yield stress [24]. This is consistent with the results of the present study, in which CAR gels showed a clear yield point.

### 2.4. pH-Indicator

**Figure 2:**
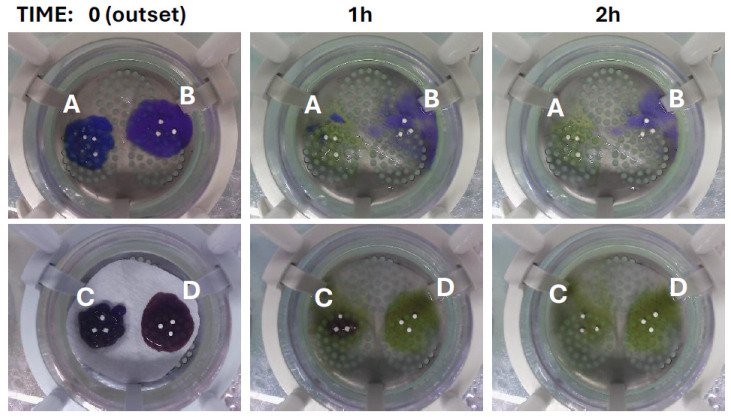
Time-dependent visual changes in selected gels containing a pH indicator during 2 h exposure to hydrochloric acid. A—22% HPMC 606; B—4% SA; C—1% CAR (NaOH); D—5% HPMC 65SH4000.

After incorporation of bromophenol blue, the selected gels showed blue to blue–burgundy coloration, depending on the formulation, which was consistent with their initial pH values ranging from mildly acidic, approximately pH 4.2, to near-neutral, approximately pH 7.0. Therefore, the initial color should be interpreted as indicating that the gel pH was above, or close to, the transition range of bromophenol blue. Upon exposure to hydrochloric acid, clear differences in the rate of color transition were observed between the formulations. Representative photographs taken during the observation period are shown in Figure 2. HPMC- and CAR-based gels underwent a rapid change from blue/blue–burgundy to yellow, indicating efficient penetration of the acidic medium into the gel matrix and acidification to below the acidic transition range of the indicator. In HPMC gels, almost the entire gel volume changed color after 1 h, suggesting rapid hydration, structural loosening, and limited resistance to medium penetration. From the perspective of enteric-coated minitablets, this behavior may be advantageous, as rapid gel dispersion is expected to facilitate the release of minitablets from the carrier and allow direct contact with the dissolution medium.

In contrast, the SA gel showed a markedly slower color transition. After 1 h, most of the sample remained blue, and even after 2 h, part of the gel retained its initial coloration. This suggests that alginate formed a more compact and less permeable matrix, which limited penetration of the acidic medium and delayed acidification of the gel interior. This behavior may be related to acid-induced gel strengthening of alginate. In an acidic medium, protonation of alginate carboxylate groups and partial formation of alginic acid reduce electrostatic repulsion between polymer chains and promote interchain association, leading to a denser and less permeable gel matrix. This compact structure may limit further acid penetration and prolong retention of minitablets within the gel carrier.

### 2.5. Pharmacopoeial Dissolution Tests

Pharmacopeial dissolution testing was performed as described in Section 4.4.1. According to European Medicines Agency (EMA) guidance for gastro-resistant dosage forms, dissolution testing should include an acid stage demonstrating gastric resistance, with less than 10% of pantoprazole dissolved after 2 h, followed by testing in a pH 6.8 buffer medium to confirm release of the majority of the pantoprazole [25].

**Figure 3:**
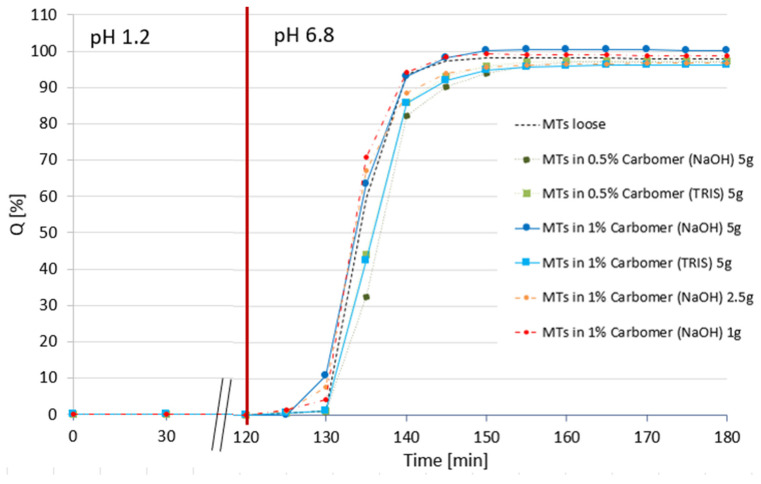
Pharmacopoeial dissolution tests for MTs dispersed in carbomer hydrogels.

The initial analysis focused on MTs dispersed in 1% CAR gels neutralized with either TRIS or NaOH. As shown in Figure 3, no pantoprazole release was observed during the acid stage, indicating that the enteric coating maintained its acid-resistant properties in the presence of CAR gels despite the relatively high pH value of 6.9. After transfer to the buffer stage, pantoprazole release was rapid and comparable to that observed for loose MTs. At least 80% of pantoprazole was released within 20 min after the change of dissolution medium, irrespective of the neutralizing agent used. Some variability was observed at the beginning of the release phase after transfer to the buffer medium; however, it remained within an acceptable range and did not affect the interpretation or comparative assessment of the dissolution profiles. Additional testing was performed using different amounts of CAR gel. For 1% CAR neutralized with NaOH, pantoprazole release was evaluated after mixing the MTs with 1.0 g, 2.5 g, and 5.0 g of gel. No marked differences in dissolution behavior were observed between the tested gel masses, indicating that reducing the amount of CAR gel did not substantially modify pantoprazole release under the applied pharmacopoeial conditions. A similar pattern was observed for the 0.5% CAR gel, further supporting that CAR gels did not markedly interfere with the release of pantoprazole from enteric-coated MTs after transfer to the buffer medium. From an application perspective, CAR gels showed favorable rheological properties. Their high apparent viscosity at rest and the presence of a flow point may help maintain a cohesive, spoonable structure and reduce the risk of leakage, spilling, or sedimentation of dispersed MTs before administration. At the same time, the marked decrease in viscosity under shear may facilitate spreading, mixing, and swallowing during oral application. Therefore, CAR gels appear to combine adequate structural stability at rest with easier flow under applied mechanical stress, which is desirable for semisolid oral vehicles intended for the administration of multiparticulate dosage forms [26].

**Figure 4:**
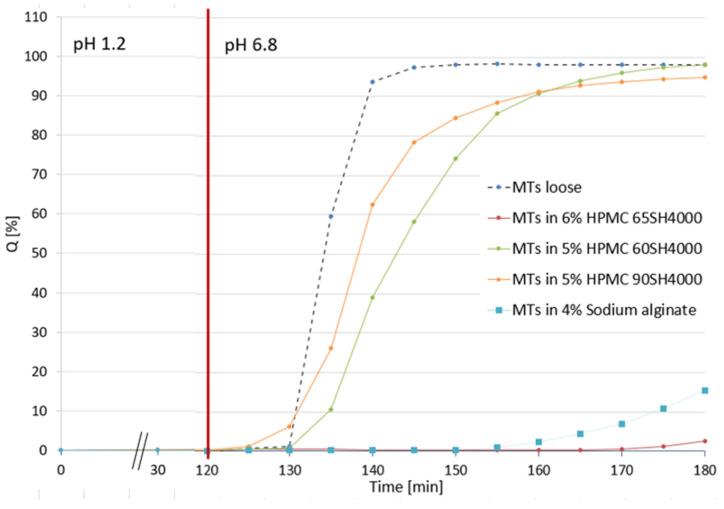
Pharmacopoeial dissolution profiles of enteric-coated MTs in HPMC and alginate gels with delayed release.

Following the analysis of CAR gels, attention was directed to gel vehicles that delayed pantoprazole release in the buffer stage relative to loose MTs. As shown in Figure 4, 5% HPMC gels of the 60SH4000 and 90SH4000 grades caused only a slight delay in drug release. In these systems, after the change of dissolution medium, more than 80% of the pantoprazole was released within 35 min, whereas for loose MTs the same threshold was reached within 20 min. Overall, the observed variability remained acceptable and did not compromise the interpretation or comparative assessment of the dissolution profiles. Although the highest SD values were observed during the dynamic release phase, particularly between 135 and 155 min for HPMC 60SH4000 and between 130 and 155 min for HPMC 90SH4000, these deviations were transient and did not affect the overall evaluation of the formulations.

The slight delay in pantoprazole release observed for 5% HPMC 60SH4000 and 90SH4000 should not be attributed to viscosity alone. Although these gels showed moderate apparent viscosity under dissolution-test conditions at 37 °C, their effect on pantoprazole release may also be related to polymer concentration and substitution-dependent gel structure.

The effect of HPMC gels on pantoprazole release was strongly concentration-dependent, but the threshold concentration at which release retardation became evident differed between HPMC grades. As demonstrated later in the dissolution study, none of the tested 4% HPMC gels markedly affected pantoprazole release. However, increasing the polymer concentration to 5% was sufficient to produce a slight delay for HPMC 60SH4000 and 90SH4000, whereas 5% HPMC 65SH4000 still did not affect the dissolution profile. For HPMC 65SH4000, a marked inhibitory effect was observed only at 6%, indicating a higher concentration threshold for the formation of a restrictive gel barrier (Figure 4). This suggests that the release-modifying effect of HPMC depends not only on the total polymer concentration but also on substitution-dependent hydration and gel-network organization. In the case of HPMC 60SH4000 and 90SH4000, increasing the concentration from 4% to 5% may have been sufficient to increase chain entanglement, local gel cohesion, and diffusional resistance around the MTs, resulting in delayed medium access to the enteric coating. HPMC 60SH and 90SH grades may form more heterogeneous or structurally compact gel layers, which can transiently hinder medium access to the enteric-coated minitablets and delay drug release. In contrast, 6% HPMC 65SH4000 likely exceeded a critical polymer concentration threshold, resulting in the formation of a persistent gel barrier that markedly limited medium penetration and pantoprazole diffusion. Notably, the gel mass remained visible in the baskets even after 2 h in the acid stage and was still present in small amounts at the end of the dissolution test, indicating high structural persistence throughout the experiment. Therefore, the observed dissolution behavior indicates grade-specific concentration thresholds rather than a simple linear relationship between apparent viscosity and drug release [27].

Overall, these findings suggest that the impact of HPMC gels on pantoprazole release from enteric-coated MTs depends on a combination of polymer concentration, substitution type, hydration behavior, and gel microstructure. Although HPMC gels may be useful as carriers for MTs, excessively concentrated systems may form persistent hydrated barriers that impair drug release and are therefore unsuitable for the administration of enteric-coated pantoprazole MTs.

**Figure 5:**
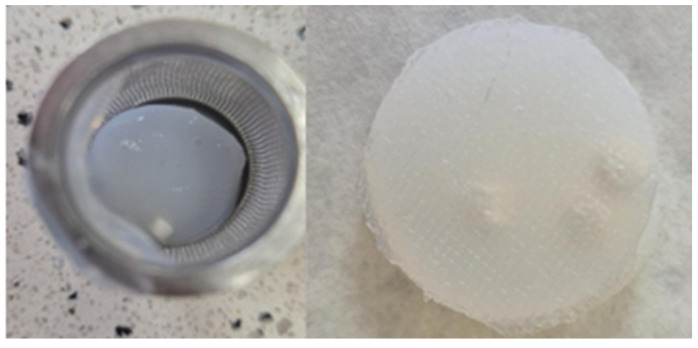
MTs in the 4% sodium alginate gel after 3 h of dissolution test.

Consistent with the pH-indicator observations, the SA gel showed the strongest tendency to hinder drug release, which may be attributed to its compact and less permeable structure, limiting medium penetration and delaying the release of enteric-coated minitablets from the gel carrier. Despite its relatively low viscosity, the 4% SA gel (Figure 4) substantially retarded drug release and failed to meet the pharmacopoeial requirements for gastro-resistant dosage forms. The behavior of SA in the two-stage dissolution test can be explained by its pH-responsive properties. In the acidic stage, protonation of SA carboxyl groups leads to shrinkage and densification of the gel network, which helps maintain structural integrity and limits swelling. After transfer to phosphate buffer, the gel becomes more hydrated and progressively loses its integrity because phosphate ions promote the release of Ca^2+^ from the calcium-alginate network, leading to swelling and eventual disintegration [28]. However, despite this pH-dependent response, drug release in the buffer stage remained markedly delayed. A photograph of the minitablets embedded in the SA gel after 3 h of dissolution testing is shown in Figure 5. Therefore, although the SA gel provided protection in the acidic stage, it was not considered a suitable vehicle for the administration of pantoprazole minitablets.

**Figure 6:**
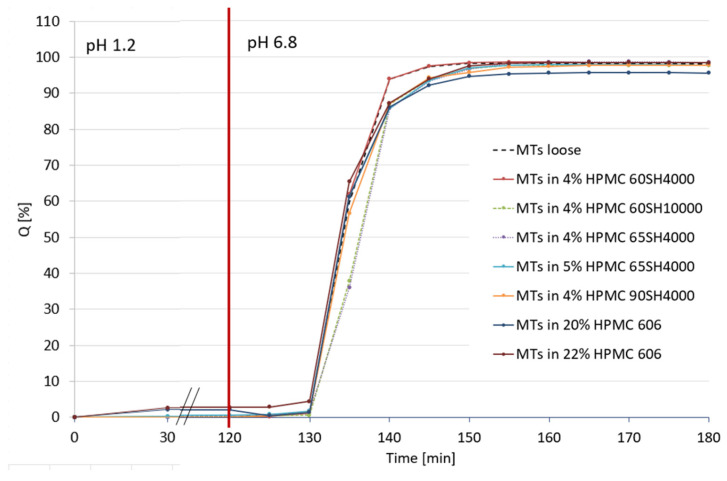
Pharmacopoeial dissolution profiles of enteric-coated MTs dispersed in selected HPMC hydrogels that did not exert a marked effect on pantoprazole release.

Figure 6 presents the dissolution profiles of enteric-coated MTs dispersed in gel vehicles that did not markedly affect pantoprazole release. As shown, all tested gels in this group allowed rapid pantoprazole release after transfer to the buffer stage. More than 80% of pantoprazole was released within 20 min after the change of dissolution medium, which was comparable to the profile observed for loose MTs. The variability of the results remained low and acceptable, with an increased SD observed only at 135 min, both for loose MTs and for MTs dispersed in gels. These transient deviations did not affect the interpretation of the dissolution profiles, as complete or near-complete drug release was subsequently achieved.

Irrespective of the HPMC substitution type, gels at a concentration of 4% did not markedly affect pantoprazole release from enteric-coated minitablets, yielding dissolution profiles comparable to those of loose MTs. In contrast, higher polymer concentrations (5% and 6%) were generally associated with delayed drug release, as shown in Figure 4. The only exception was 5% HPMC 65SH4000, for which no noticeable impact on the dissolution profile was observed, in contrast to the slight delay observed for 60SH4000 and 90SH4000, which may therefore be attributed to differences in polymer substitution type and, consequently, hydration kinetics and gel microstructure. It is plausible that HPMC 65SH4000 forms a more homogeneous and rapidly hydrated gel network, allowing efficient penetration of the dissolution medium and limited diffusional resistance.

Although HPMC 606 provided very favorable dissolution results, its practical applicability as an oral gel vehicle should be considered with caution. Gels based on this polymer required concentrations above 20% to achieve suitable application properties, which makes this approach less economical and potentially difficult to justify from a formulation-development perspective. Moreover, a pronounced increase in viscosity was observed at 37 °C, indicating that the gel may behave unpredictably under administration and dissolution-test conditions. This temperature-dependent viscosity increase may also reduce batch-to-batch reproducibility and complicate process control. Therefore, despite its promising dissolution profile, HPMC 606 may be less suitable as a routine gel vehicle than other HPMC grades, which produced comparable release behavior at substantially lower polymer concentrations.

### 2.6. Advanced Dissolution Tests

**Figure 7:**
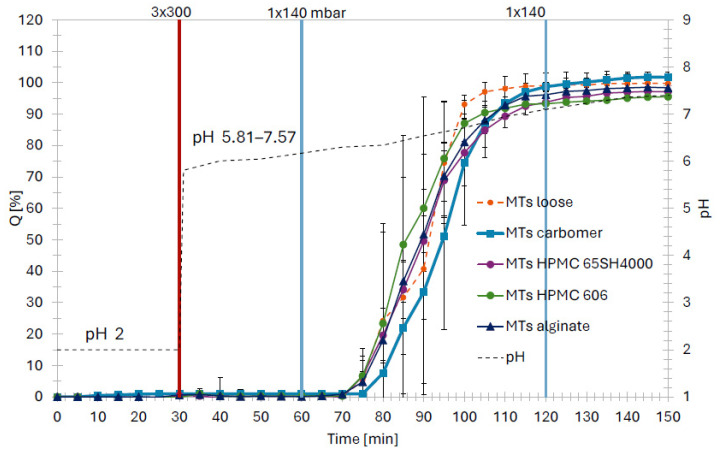
Non-pharmacopoeial dissolution profiles of enteric-coated MTs dispersed in selected gel vehicles using the AMP.

The release profiles obtained under non-pharmacopoeial dynamic Advanced Modular Platform (AMP) conditions are presented in Figure 7. In this method, after 30 min of exposure to the acidic phase, gastric emptying and passage through the pylorus were simulated by three contraction cycles of 300 mbar. A buffer concentrate was then added to the acceptor medium, increasing the pH to approximately 5.81, followed by a gradual rise to pH 7.57. After the pH increase, the loose MTs showed complete dissolution, with more than 80% of pantoprazole released within approximately 70 min. A similar release profile was observed for MTs administered in 22% HPMC 606 gel, indicating that this vehicle did not markedly delay pharmaceutical availability under the applied dynamic conditions. This observation is consistent with the pharmacopoeial two-stage dissolution test, in which HPMC 606 allowed rapid pantoprazole release after transfer to the buffer stage. Similarly, for MTs administered in 5% HPMC 65SH4000 gel, more than 80% pantoprazole release was recorded approximately 75 min after the pH increase, with a profile close to that of loose MTs. Under dynamic AMP conditions, the HPMC 606 and HPMC 65SH4000 gels were sufficiently penetrated or dispersed to allow effective liberation of the enteric-coated MTs and pantoprazole release.

The relatively large SD values observed between approximately 70 and 100 min are not unexpected for this type of dynamic dissolution experiment and reflect variability during the transitional phase of the AMP test. At this stage, pH elevation, mechanical stress events, gel disruption, liberation of MTs from the gel vehicle, and onset of enteric-coating dissolution occur in close temporal sequence. Consequently, small differences in the timing of MT liberation or coating dissolution between individual vessels may result in larger variability at these time points. The AMP experiments were performed in triplicate, as this part of the study was intended as an exploratory comparative assessment supporting formulation screening rather than as a final release-testing procedure.

The most notable difference between the pharmacopoeial and non-pharmacopoeial tests was observed for the SA gel. In the two-stage pharmacopoeial dissolution test, 4% SA gel markedly retarded pantoprazole release and failed to meet the dissolution requirements for gastro-resistant dosage forms. However, under dynamic AMP conditions, SA did not cause a relevant delay in drug release, with more than 80% of pantoprazole released by approximately 75 min after the pH increase. Thus, the SA gel showed method-dependent behavior, with marked retardation under pharmacopoeial conditions but no relevant delay under the applied dynamic AMP conditions.

Finally, CAR gels did not show pantoprazole release during the acidic stage or during the simulated gastric emptying phase. No initial release was observed despite the application of pressure events and the subsequent increase in medium pH, indicating that the enteric coating maintained its integrity in the presence of CAR gels under the applied dynamic AMP conditions. After the pH transition and disruption of the gel structure, the liberated MTs showed rapid pantoprazole release, with dissolution profiles comparable to those of loose MTs. Following the pH increase, the main release phase began, and at least 80% of pantoprazole release was recorded after approximately 75 min, when the pH of the medium reached approximately 7.0. These findings suggest that CAR gels did not compromise the acid resistance of the enteric-coated MTs and, after mechanical disruption and pH transition, allowed effective liberation of the MTs and subsequent drug release.

The observed differences between hydrogel vehicles can be explained by the combined effects of polymer concentration, gel-network structure, medium penetration, and polymer responsiveness to pH and mechanical stress. In HPMC gels, which are non-ionic cellulose ether systems, the effect on dissolution may be associated with hydration behavior, polymer-chain entanglement, and the possible formation of a hydrated diffusional barrier around the dispersed MTs. At lower concentrations, the gel network was sufficiently hydrated and dispersible to allow rapid penetration of the dissolution medium and effective liberation of the enteric-coated MTs. In contrast, higher HPMC concentrations likely increased network density and tortuosity, thereby slowing medium access to the minitablets and delaying drug release. The differences observed between HPMC grades of similar nominal viscosity suggest that apparent viscosity alone does not fully determine diffusion behavior. These differences may be related to substitution-dependent hydration kinetics and gel-network characteristics; however, because direct structural characterization of hydrated HPMC gels was not performed, this interpretation should be regarded as a mechanistic hypothesis supported by rheological and dissolution data rather than as direct evidence of microstructural differences. For CAR gels, the mechanism is different and is primarily related to the pH-responsive behavior of crosslinked polyacrylic acid. This may explain why CAR gels maintained acid-stage protection but allowed rapid pantoprazole release after the pH transition. SA, in turn, may form a comparatively compact and cohesive matrix under acidic conditions due to reduced ionization and acid-induced structural contraction, which can limit medium penetration under static pharmacopoeial conditions.

The discrepancy observed for SA between the pharmacopoeial and AMP dissolution tests is mechanistically and practically important. Under pharmacopoeial conditions, the SA gel was exposed to a more acidic medium, 0.1 M HCl, for a longer period of 2 h, whereas the AMP test used 0.01 M HCl at pH 2.0 for 30 min, followed by a dynamic pH transition. The stronger and longer acid exposure in the pharmacopoeial test may have promoted more extensive protonation of alginate carboxylate groups and the formation of a compact alginic-acid-rich matrix, thereby reducing gel permeability, limiting medium penetration, and delaying the liberation of MTs from the gel vehicle. In contrast, under AMP conditions, the lower acid strength, shorter gastric exposure, gradual pH increase, and mechanical stress events simulating gastric emptying and intestinal pressure events probably disrupted or loosened the alginate matrix and reduced its barrier effect. Therefore, the favorable behavior of SA under AMP conditions should be interpreted as evidence that dynamic gastrointestinal conditions may partially overcome the barrier observed in the static pharmacopoeial test, rather than as sufficient evidence of formulation suitability. From a practical and regulatory perspective, this discrepancy is critical. Pharmacopoeial dissolution testing remains the primary quality-control and release-testing framework for gastro-resistant dosage forms; therefore, failure to meet pharmacopoeial dissolution requirements represents a major limitation, even if the formulation performs more favorably under biorelevant dynamic conditions. AMP testing may provide valuable mechanistic and biopredictive insight, but it should be regarded as complementary to, rather than a replacement for, compendial dissolution testing. These observations confirm that hydrogel vehicles for enteric-coated multiparticulates should be evaluated not only in terms of bulk viscosity and handling properties, but also with respect to hydration, pH responsiveness, gel-network persistence, medium penetration, and mechanical disruption under gastrointestinally relevant conditions.

It should be emphasized that the gel systems evaluated in this study were intended as administration vehicles, with enteric-coated pantoprazole MTs mixed with the gel immediately before administration. Given the moisture sensitivity of pantoprazole and the high water content of hydrogels, such studies would be required if these systems were to be developed as ready-to-use formulations.

A further limitation of this study is that the proposed mechanisms related to gel-network structure and possible gel–coating interactions were inferred mainly from rheological behavior, pH-indicator observations, visual assessment, and dissolution performance. Representative microscopic images of the coated MTs and coating cross-sections are provided in Supplementary Figure S1; direct characterization of the hydrated gel microstructure and molecular or surface-level interactions between the hydrogel vehicles and the enteric coating was not performed. Therefore, terms such as gel-network organization, matrix compactness, and gel–coating compatibility should be interpreted as mechanistic hypotheses supported by indirect experimental evidence. Future studies should include complementary structural and surface-sensitive methods, such as microscopy of hydrated gels, imaging of MTs after contact with gel vehicles, and spectroscopic analysis of potential polymer–coating interactions.

In addition, the study should be interpreted in the context of its exploratory formulation-screening design. The number of replicates, including the use of three independent vessels in dissolution testing, was selected to identify formulation-dependent trends and compare the behavior of candidate gel vehicles rather than to provide a fully powered inferential comparison or formal pharmacopoeial batch-release assessment.

## 3. Conclusions

This study demonstrated that appropriately selected oral hydrogel vehicles may support the administration of enteric-coated pantoprazole MTs, but their impact on gastro-resistance and drug release must be carefully evaluated. Among the tested systems, low-concentration gels prepared from high-viscosity HPMC grades showed the most favorable overall performance. In particular, 5% HPMC 65SH4000 combined suitable application properties with limited influence on pantoprazole release in both pharmacopoeial and dynamic AMP dissolution tests.

CAR gels also showed formulation potential because of their favorable spoonable consistency, high viscosity at rest, yield-stress behavior, and ability to allow rapid pantoprazole release after pH transition. However, because CAR gels require neutralization and may interact with pH-dependent enteric coatings, their composition and pH should be carefully controlled. In contrast, SA gel showed marked retardation of pantoprazole release under pharmacopoeial conditions and therefore cannot be considered a suitable vehicle for enteric-coated pantoprazole MTs without further optimization, despite more favorable behavior under dynamic AMP conditions.

Overall, hydrogel vehicles should not be regarded as neutral carriers for enteric-coated multiparticulates. Their polymer type, concentration, pH, rheological behavior, and matrix properties may influence medium penetration, liberation of MTs, and subsequent drug release. Therefore, selection of gel vehicles for gastro-resistant multiparticulate formulations should be based not only on handling and swallowability, but also on demonstrated compatibility with the enteric coating under relevant dissolution conditions.

## 4. Materials and Methods

### 4.1. Production and Characterization of Uncoated Minitablets

The MT cores were composed of 25% *w*/*w* pantoprazole sodium sesquihydrate (Hetero Drugs, Hyderabad, India), microcrystalline cellulose (Vivapur PH102, JRS Pharma, Rosenberg, Germany), dicalcium phosphate (Di-Cafos A 150, Budenheim KG, Budenheim, Germany), croscarmellose sodium (Ac-Di-Sol, FMC BioPolymer, Philadelphia, PA, USA), and sodium stearyl fumarate (PRUV, FMC BioPolymer, Newark, DE, USA). Pantoprazole sodium was granulated in a high-shear wet granulator (Prymus, Zelmer, Rzeszów, Poland) using a 25% *w*/*w* sodium carbonate solution (PPH STANLAB, Lublin, Poland). Biconvex MTs with a diameter of 3 mm and a mean weight of 22.5 mg were prepared using a compression force of 14 kN on a rotary tablet press (RTP-D8, Erweka, Heusenstamm, Germany) equipped with a multi-tip (13×) tooling system. The crushing resistance of the MT cores (*n* = 20) was evaluated using a TA.XT Plus texture analyzer (Stable Micro Systems, Godalming, UK). Friability testing was performed according to the Ph. Eur. 12.1 (Chapter 2.9.7). Previously dedusted MTs (6.5 g) were placed in a friability tester (TAR-10, Erweka, Heusenstamm, Germany), and after 100 rotations, the mass loss was determined.

### 4.2. Production and Characterization of Enteric-Coated Minitablets

A 4M8-Trix fluid-bed apparatus (ProCepT, Zelzate, Belgium) was used for the laboratory-scale coating of MTs in 50 g batches. First, the MTs were subcoated using a 10% *w*/*w* HPMC (Pharmacoat 606, Shin-Etsu Chemical, Tokyo, Japan) solution containing PEG 6000 (Sigma-Aldrich, Steinheim, Germany) in a 9:1 ratio. The enteric layer was applied using Eudragit L 30D-55 (Evonik Industries, Darmstadt, Germany), a methacrylic acid–ethyl acrylate copolymer (1:1) with pH-dependent solubility above 5.5. The aqueous coating dispersion, containing 20% solids, was prepared according to the manufacturer’s recommendations. Triethyl citrate (Sigma-Aldrich, Steinheim, Germany) was added as a plasticizer, talc (Luzenac VAL Chisone, Porte, Italy) as an anti-adherent, and water was added to the Eudragit L dispersion. The coating process conditions are presented in Table 2. The coating thickness was determined microscopically using cross-sections of sampled MTs and a VHX-7000 digital microscope (Keyence, Osaka, Japan). Samples were examined under reflected light. Mean coating thickness was calculated from measurements taken at 10 positions on each cross-section in at least five MTs. Representative microscopic images of the enteric-coated pantoprazole minitablets, including the external appearance and cross-sectional view with coating thickness measurement, are provided in Supplementary Figure S1.

Disintegration testing was performed in accordance with Ph. Eur. 12.1 requirements for gastro-resistant dosage forms (Chapter 2.9.1). Enteric-coated MTs were kept in 0.1 mol/L HCl for 2 h and then transferred to phosphate buffer (pH 6.8). The time to disintegration after medium replacement was recorded. Because of the small size of the MTs, the standard mesh at the bottom of the basket-rack assembly was replaced with a finer mesh to prevent intact minitablets from passing through the screen before disintegration [29]. The endpoint was verified visually.

### 4.3. Production and Characterization of Oral Gel Vehicles

Oral gels were prepared in an aqueous medium according to the manufacturers’ instructions for the respective polymers. The polymers were selected to represent pharmaceutically established gel-forming systems with distinct structural and functional properties. HPMC was included as a non-ionic cellulose ether widely used in oral pharmaceutical formulations, with viscosity grades allowing modulation of gel consistency and limited ionic or pH-dependent interactions. Carbomer was selected as a crosslinked polyacrylic acid polymer forming structured, shear-thinning, yield-stress gels after neutralization, which may be advantageous for spoon dosing and retention of dispersed MTs before administration. Sodium alginate was included as a natural anionic polysaccharide with ion- and pH-responsive gelation behavior, making it relevant for evaluating the influence of gastric-to-intestinal pH transition on gel structure and MT liberation. These polymers were therefore chosen to compare three distinct hydrogel systems differing in polymer origin, charge, pH responsiveness, rheological behavior, and potential interaction with enteric-coated MTs.

#### 4.3.1. Carbomer Hydrogels

CAR (Carbopol 974P NF, Lubrizol, Wickliffe, OH, USA) hydrogels at concentrations of 0.5% and 1.0% were prepared. The required amounts of polymer were dispersed in purified water under magnetic stirring, and the resulting dispersions were left until complete hydration and swelling. Subsequently, the CAR was neutralized with either a 10% sodium hydroxide solution or a 20% TRIS solution, resulting in gelation and hydrogel formation.

#### 4.3.2. Hypromellose Hydrogels

HPMC hydrogels were prepared from selected Shin-Etsu Chemical (Tokyo, Japan) grades differing in pharmacopoeial type and manufacturer-declared viscosity (Table 3), at concentrations specified in Table 1. Appropriate amounts of HPMC were dispersed in hot purified water under stirring. Upon cooling, the polymers hydrated and dissolved, forming homogeneous hydrogels.

#### 4.3.3. Sodium Alginate Hydrogel

A 4% SA hydrogel was prepared by dispersing the required amount of sodium alginate (Agnex, Białystok, Poland) in purified water using a high-speed stirrer. After complete hydration and dissolution of the polymer, the stirring speed was increased to the maximum and 5% CaCl_2_ was added to crosslink the polymer, resulting in hydrogel formation.

#### 4.3.4. pH Measurement

The pH of the prepared gels was measured potentiometrically using a pH meter equipped with a glass combination electrode (Orion Research, Model 350, Boston, MA, USA), with the electrode directly immersed in each gel sample.

#### 4.3.5. Analysis of Rheology and Texture of Hydrogels

Rheological properties of the gels were investigated using a rotational rheometer (Anton Paar MCR 102e, Graz, Austria) equipped with a cone–plate geometry. The cone had a diameter of 25 mm, an angle of 1°, and a measuring gap of 0.047 mm. For data analysis, Anton Paar RheoCompass 1.35 was used. For each measurement, approximately 0.2 mL of gel was applied and equilibrated for 2 min under thermostated conditions at 25 ± 0.1 °C or 37 ± 0.1 °C before analysis. Flow curves were obtained over a shear-rate range of 0.01–500 s^−1^ by recording shear stress and apparent viscosity as functions of shear rate. For inter-formulation comparison, apparent viscosity values at 0.01 and 100 s^−1^ were used (*n* ≥ 3). The results are presented as mean values from at least three measurements with the corresponding SD.

Oscillatory amplitude sweep tests were performed at a constant angular frequency of ꙍ = 10 rad/s under strain-controlled conditions, within a shear strain range of 0.1–500%. For each measurement, approximately 0.2 mL of sample was applied to the fixed lower plate of the rheometer and equilibrated for 2 min before analysis. To characterize viscoelastic behavior, the storage modulus (G′) and loss modulus (G″) were recorded during rheological measurements performed in triplicate for each formulation. The flow point was determined as the crossover point at which G′ = G″. The storage modulus (G′) was used as an indicator of the elastic component, whereas the loss modulus (G″) represented the viscous component of the hydrogel response.

Texture analysis was performed using a TA.XT Plus Texture Analyzer (Stable Micro Systems, Godalming, UK) equipped with an A/BE back-extrusion ring probe (Ø 40 mm) and Texture Exponent software (SET19002, Stable Micro Systems, Godalming, England). The test parameters were as follows: pre-test speed, 15 mm/s; test and post-test speeds, 2.0 mm/s; penetration depth, 5 mm; and trigger force, 10 g [30]. Each hydrogel sample was analyzed in triplicate at ambient temperature, with empirically selected 1 h intervals between consecutive measurements to allow recovery of the gel structure. Firmness values are presented in Table 1 as mean ± SD.

#### 4.3.6. pH-Indicator

Bromophenol blue was used as the pH indicator, as it changes color from yellow under acidic conditions below approximately pH 3.0 to blue at pH values above 4.6. The pH indicator was introduced into the gel vehicles as a methanol solution (5% *w*/*v*) to obtain a final concentration of approximately 0.65% *w*/*v*. Briefly, 1.5 mL of the indicator solution was mixed with approximately 10 mL of each gel until homogeneous distribution was achieved. The gel containing the pH indicator was then placed on a perforated plate and immersed in hydrochloric acid solution at pH 2.0 to a depth allowing only the bottom surface of the gel to remain in contact with the acid. The experiment was conducted for 2 h, during which photographs of the gels were taken every 2 min and subsequently assessed visually. The color transition from blue to yellow was used as a qualitative marker of hydrochloric acid penetration into the gel matrix and the progressive acidification of the formulation.

### 4.4. Release of the Pantoprazole from Minitablets

#### 4.4.1. Pharmacopoeial Dissolution Tests

In vitro dissolution testing was conducted using the Ph. Eur. basket apparatus (DT 720 Series, Erweka, Heusenstamm, Germany) at 100 rpm and 37.0 ± 0.5 °C. MTs were tested as loose units or after incorporation into 5 g of gel. Additional tests were performed for 1% CAR gel neutralized with NaOH using reduced gel amounts of 2.5 g and 1 g. Loose enteric-coated MTs were used as the internal reference, as the primary objective was to assess the effect of hydrogel vehicles on the dissolution behavior of the same developed multiparticulate formulation. Each test sample contained four MTs, corresponding to 20 mg of pantoprazole per vessel, and was first tested in 900 mL of 0.1 M HCl. After 2 h, the medium was replaced with 900 mL of potassium phosphate buffer (pH 6.8), and dissolution testing was continued for 1 h. Pantoprazole release was monitored online using a UV–Vis spectrometer equipped with flow-through spectrophotometric cuvettes (Agilent, Santa Clara, CA, USA), at 305 nm during the acid phase and 288 nm during the buffer phase. The mean amount of released pantoprazole (*n* = 3) was calculated using calibration curves prepared in the respective media; the analytical method was validated. The use of three independent vessels per formulation was selected for this exploratory formulation-screening stage, which aimed to compare the influence of gel vehicles on dissolution behavior rather than to perform formal batch-release or regulatory quality-control testing. Compliance with the Ph. Eur. 12.1 requirements for gastro-resistant dosage forms was assessed according to Chapter 2.9.3.

#### 4.4.2. Advanced Dissolution Tests

##### Apparatuses

**Figure 8:**
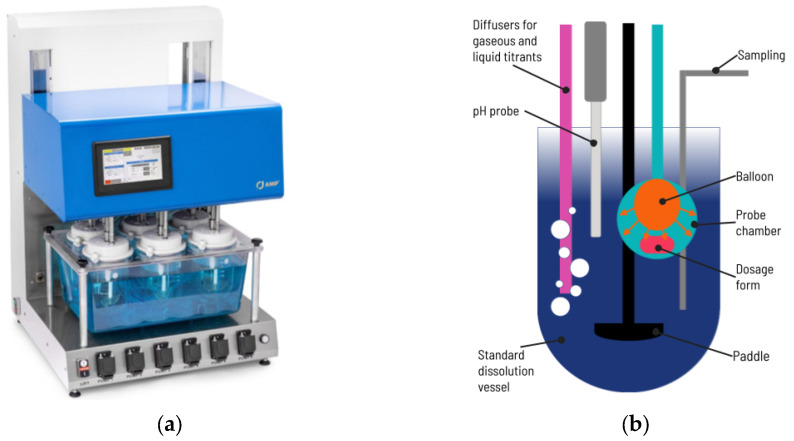
Schematics of the AMP apparatus: image of the AMP apparatus (**a**), stress test configuration (**b**).

Dissolution tests were conducted using the Advanced Modular Platform (AMP, Figure 8a), a novel apparatus developed by Physiolution [31]. It is designed to simulate biorelevant pH gradients, physiological mechanical stresses (50−550 mbar) and movement of the dosage form (5−50 mm/s) in the gastrointestinal tract. AMP is equipped with vessels and paddles typical for a USP 2 apparatus; however, the dosage form can be placed either at the bottom of the vessel or in the probe chamber, where it can be subjected to stress events caused by inflation of an elastic balloon (stress test configuration—Figure 8b). The pH of the medium is constantly monitored by a pH electrode installed inside the vessel and can be automatically adjusted by adding relevant titrants (acidic or alkaline). Sampling can be performed automatically or manually. Dissolution tests of MTs dispersed in the gels were performed using the AMP in the stress test configuration.

##### Test Solutions

As a medium intended to simulate the fasted gastric conditions, 900 mL of 0.01 M HCl pH 2.0 was used. The pH change at the simulated gastric emptying was performed by an automated addition of 100 mL of NaHCO_3_ solution containing 2.3 g of 3F powder (Biorelevant, Ltd., London, UK), which resulted in a Fasted-State Simulated Intestinal Fluid (FaSSIF) concentration of physiological surfactants and buffering capacity of 5 mmol/ΔpH/L.

##### Test Conditions

The physicochemical and mechanical robustness of the minitablets and the gel vehicles was evaluated during dissolution tests by simulating multiple high-intensity stress events and dynamic gradients of pH. For this purpose, the gel vehicles were introduced gravimetrically into the probe chambers, then a defined number of minitablets was added and gently mixed with the gel to achieve complete immersion. Subsequently, the baskets were assembled on the device, and the tests were started.

##### Dissolution Testing Under Simulated Fasted Conditions

Dissolution performance was evaluated using AMP in a stress test configuration that was intended to simulate the fasted intake conditions. Dissolution tests were performed in two stages, with the addition of buffer concentrate between the gastric and intestinal stages. In both test scenarios, MTs were subjected to a series of mechanical stresses in the form of pressure events and elevated movement speed. These events reproduced the mechanical conditions experienced by the dosage form during passage through the pylorus, i.e., gastric emptying (GE), and through the proximal small intestine, i.e., intestinal pressure events (IntPE). A detailed description of the test conditions is presented in Table 4. Each test was performed using three replicates. The samples were filtered through polyethylene filters (1-micron full-flow filters, UHMW PE, Dissolution Accessories, ProSense B.V., Oosterhout, The Netherlands) and analyzed using online UV-Vis spectroscopy in closed-loop mode (Agilent 8453, Paolo Alto, CA, USA).

### 4.5. Data Analysis

Data are presented as mean ± SD. The study was designed as an exploratory formulation-screening study; therefore, no formal inferential statistical comparisons between all formulations were performed. The interpretation of rheological, texture, and dissolution data was based on descriptive comparisons, pharmacopeial dissolution criteria, and consistency of formulation-dependent trends across replicates.

## Figures and Tables

**Table 1:** Physicochemical characterization of oral gel vehicles.

Polymer		pH	Viscosity [Pa·s] (*n* ≥ 3 ± SD)				Firmness [g] (*n* ≥ 3 ± SD)
Type and Concentration [%]			Shear Rate 25 °C		Shear Rate 37 °C		
			0.01 [s^−1^]	100 [s^−1^]	0.01 [s^−1^]	100 [s^−1^]	
SODIUM ALGINATE (SA)	4	7.2	40.8 ± 2.5	6.8 ± 0.3	32.5 ± 2.2	5.5 ± 0.1	112.7 ± 2.0
CARBOMER (CAR) + NaOH	0.5	6.3	1524.5 ± 141.8	2.0 ± 0.02	1293.8 ± 180.2	2.0 ± 0.2	167.3 ± 3.8
	1	6.9	2403.5 ± 64.2	3.4 ± 0.1	1537.5 ± 219.3	3.3 ± 0.1	231.5 ± 11.0
CARBOMER (CAR) + TRIS	0.5	6.2	1661.00 ± 60.4	1.9 ± 0.02	853.6 ± 55.0	1.9 ± 0.1	160.8 ± 5.7
	1	6.0	2321.5 ± 37.7	3.5 ± 0.1	1841.4 ± 339.4	3.2 ± 0.1	215.7 ± 19.8
HPMC 60SH4000	4	5.3	69.8 ± 3.6	6.3 ± 0.2	83.0 ± 36.2	5.0 ± 0.3	152.3 ± 5.7
	5	4.5	181.0 ± 1.2	11.9 ± 0.1	117.4 ± 13.5	9.2 ± 0.3	252.9 ± 3.4
HPMC 65SH4000	4	4.3	83.5 ± 9.1	7.9 ± 0.6	72.1 ± 16.6	7.5 ± 0.5	163.5 ± 0.4
	5	4.2	169.1 ± 22.3	12.6 ± 0.7	148.6 ± 28.00	10.9 ± 0.5	292.6 ± 3.0
	6	4.2	356.2 ± 38.6	19.9 ± 1.5	443.7 ± 39.3	21.5 ± 0.7	560.1 ± 11.7
HPMC 606	20	7.8	16.3 ± 2.9	5.8 ± 0.1	30,999.0 ± 6853.9	4.3 ± 0.3	46.4 ± 4.2
	22	7.2	29.7 ± 4.2	10.7 ± 0.3	31,106.0 ± 3117.2	8.0 ± 0.2	72.7 ± 4.3
HPMC 90SH4000	4	4.3	58.1 ± 21.5	7.7 ± 1.4	58.5 ± 8.1	6.65 ± 1.0	157.2 ± 13.9
	5	4.3	151.2 ± 12.1	13.9 ± 0.2	110.3 ± 14.5	10.9 ± 0.8	271.4 ± 36.5

**Table 2:** Process parameters for the coating of pantoprazole minitablets.

	HPMC (Subcoating)	Eudragit L 30 D-55 (Enteric Coating)
Inlet airflow	0.40 m^3^/min	0.40 m^3^/min
Inlet air temperature	65.0 °C	30.0 °C
Product temperature	45.0 °C	26.0 °C
Atomization pressure	1 bar	1 bar
Spray rate of the coating dispersion	1.1 g/min	0.9 g/min

**Table 3:** Characteristics of the HPMC grades used in the study, including manufacturer-declared viscosity of 2 wt.% aqueous solutions.

HPMC Grade	Commercial Name	Pharmacopoeial Type	Manufacturer-Declared Viscosity *
HPMC 606	Pharmacoat^®^ 606	2910	6 mPa·s (low-viscosity)
HPMC 60SH4000	METOLOSE^®^ 60SH-4000	2910	4000 mPa·s (high-viscosity)
HPMC 65SH4000	METOLOSE^®^ 65SH-4000	2906	4000 mPa·s (high-viscosity)
HPMC 90SH4000	METOLOSE^®^ 90SH-4000	2208	4000 mPa·s (high-viscosity)

* 2 wt.% aqueous solution at 20 °C, according to the USP method.

**Table 4:** Dissolution tests under simulated fasted conditions.

Parameter	Fasted
Gastric medium	0.01 M HCl, pH = 2.0, 900 mL, 30 min
Intestinal medium	FaSSIF, pH = 5.81–7.57, 1000 mL, 150 min
Paddle rotation [rpm]	50
Temperature	gradient from room temp. to 37 °C within 15 min
Probe chamber idle movement [mm/s]	5
	Gastric emptying (GE)
Pressure	3 × 300 mbar
Movement	1 min at 50 mm/s
Timing	30 min
	Intestinal pressure events (IntPE)
Pressure	1 × 140 mbar
Movement	0.5 min at 25 mm/s
Timing	1, 2, 3 h

## Data Availability

The data supporting the findings of this study are available from the corresponding author upon reasonable request.

## Supplementary Materials

The following supporting information can be downloaded at: https://www.mdpi.com/article/10.3390/gels12060526/s1, Figure S1. Representative images of pantoprazole minitablets: (a) uncoated minitablets; (b) enteric-coated minitablets; (c) microscopic cross-section of enteric-coated minitablets showing the coating layer, observed at 200× magnification.

## Abbreviations

The following abbreviations are used in this manuscript:

MTs	minitablets
HPMC	hypromellose
CAR	carbomer
SA	sodium alginate
AMP	Advanced Modular Platform
PPI	proton pump inhibitor
Ph. Eur.	European Pharmacopoeia
TRIS	tris(hydroxymethyl)aminomethane
M	β-D-mannuronic acid
G	α-L-guluronic acid
EMA	European Medicines Agency
GE	gastric emptying
IntPE	intestinal pressure events
