# Supplementary material for: Hydrogel Vehicles for Enteric-Coated Pantoprazole Minitablets: Impact of Polymer Type on Rheology and Drug Release
Source: Gels. 2026 Jun 11;12(6):526. doi: 10.3390/gels12060526 (PMC13297835; doi:10.3390/gels12060526)
Supplement: Supplementary file 1 [file gels-12-00526-s001.zip › gels-4350401-supplementary.pdf]

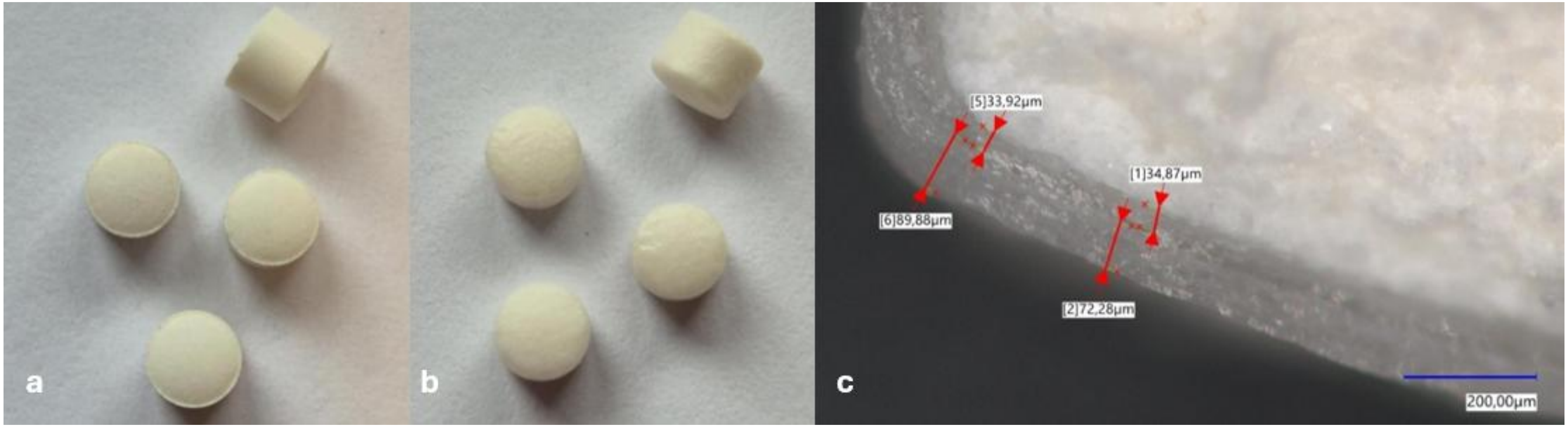

Supplementary Figure S1. Representative images of pantoprazole minitablets: (a) uncoated minitablets; (b) enteric-coated minitablets; (c) microscopic cross-section of enteric-coated minitablets showing the coating layer, observed at 200× magnification.
